# High-energy-resolution X-ray monochromator calibration using the detailed-balance principle

**DOI:** 10.1107/S0909049512022339

**Published:** 2012-06-09

**Authors:** J. Y. Zhao, W. Sturhahn

**Affiliations:** a Advanced Photon Source, Argonne National Laboratory, 9700 South Cass Avenue, Argonne, IL 60439, USA

**Keywords:** X-ray, monochromator, energy calibration, nuclear resonant scattering

## Abstract

A new method is presented to calibrate an X-ray energy scale with sub-meV relative accuracy by using the detailed-balance principle of the phonon creation and annihilation. This method is conveniently used to define or verify the energy scale of high-energy-resolution monochromators that are used in inelastic X-ray scattering and nuclear resonant inelastic X-ray scattering instruments at synchrotron radiation facilities. This method does not rely on sample properties and its precision only depends on the statistical data quality. Well calibrated instruments are essential for reliable comparison of data sets obtained at different synchrotron radiation beamlines, of data with theoretical predictions, and of data from other techniques such as neutron or light scattering. The principle of the detailed-balance method is described in this paper and demonstrated experimentally.

## Abstract 2

A sample-independent method is presented to calibrate an X-ray energy scale of a high-energy-resolution monochromator with sub-meV relative accuracy by using the detailed-balance principle.

## 1. Introduction

Owing to the tremendous increase of the X-ray brilliance at third-generation synchrotron radiation light sources in the past two decades, applications of X-rays have been broadened to many once-impossible research areas. Among them, momentum-resolved inelastic X-ray scattering (IXS) and nuclear resonant inelastic X-ray scattering (NRIXS) have become powerful tools for studying low-energy collective excitations in condensed matter that have been traditionally studied by Raman scattering, infrared absorption and neutron scattering (Krisch & Sette, 2007 ▶; Burkel, 2000 ▶; Sturhahn & Jackson, 2007 ▶; Sturhahn, 2004 ▶; Röhlsberger, 2004 ▶; Sturhahn *et al.*, 1995 ▶; Seto *et al.*, 1995 ▶). The study of phonons typically requires X-ray energy bandwidths at the meV level which are produced by the use of a high-energy-resolution monochromator (HRM) (Toellner *et al.*, 2006 ▶; Shvydko, 2004 ▶; Toellner, 2000 ▶; Burkel, 2000 ▶). The development of crystal optics with high resolution and efficiency has been essential for the rapid progress of IXS and NRIXS at various synchrotron radiation facilities. HRMs typically operate in a narrow energy range around an energy between 10 keV and 30 keV with efficiencies between 10% and 50%. The operating energy is either determined by single-crystal back-reflection (IXS) or a nuclear transition energy (NRIXS). The energy range for phonon studies is typically ±100 meV around the operating energy. Energy scanning of a HRM can be achieved either by altering lattice parameters, *e.g.* changing the temperature of a back-reflection crystal, or by altering the relative scattering angles among several crystals. The energy scale of a HRM is determined by energy–temperature or energy–angle relationships. These theoretical relationships in combination with high-precision temperature and angle measurements usually provide energy scales within a few-percent accuracy. However, the actual system response may deviate from the ideal case, *e.g.* caused by thermal gradients or slight misalignments of monochromator crystals, and for higher accuracy additional calibration is required. At present, IXS and NRIXS measurements routinely produce high-quality data, and accurate energy calibration facilitates inter-laboratory comparisons and tests of theoretical predictions. For example, a popular method for calibrating the energy scale of a HRM is to use well known phonon excitations of certain standard samples. The drawback of this calibration method is the need for a standard sample which has to be chemically stable and environmentally controlled. Furthermore, the phonon excitation energies have to be known precisely by independent measurements or calculations. It is thus very desirable to develop a sample-independent method for energy-scale calibration.

In this paper we present an energy-scale calibration method based on the detailed-balance principle of the phonon creation and annihilation in an inelastic X-ray scattering spectrum. The precision of the calibration mostly depends on the statistical quality of the data. With an IXS spectrum of good quality and an accurate sample temperature measurement, the energy scale can be calibrated using an arbitrary sample. We explain the principle of this method in this paper and demonstrate it by using the NRIXS technique. All derivations presented in the following, however, are also valid for non-resonant IXS spectra.

## 2. Principle of the method

The shape of an inelastic scattering spectrum is described by an excitation probability density *S*(ω), where ω is the energy transfer to the sample and other dependences are suppressed for clarity. Elastic scattering corresponds to ω = 0 and positive/negative values of ω describe energy transfer to/from the sample from/to the X-rays. For bosonic excitations such as phonons, the function *S*(ω) satisfies a ‘detailed balance’ given by the Boltzmann factor
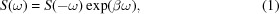
where β = 1/(*k*_B_ *T*) with temperature *T* and Boltzmann constant *k*_B_. The phonon creation/annihilation sidebands of *S*(ω) are conceptually equivalent to the Stokes and anti-Stokes lines of optical spectroscopies. We will call the rule expressed by (1) the ‘detailed-balance principle’. This has been discussed for NRIXS (Sturhahn & Kohn, 1999 ▶) but is an intrinsic feature of all inelastic phonon spectra and does not depend on the sample properties. The ratio *S*(ω)/*S*(−ω) can be determined experimentally and has been used to derive sample temperatures under extreme high pressures during laser heating in a diamond anvil cell (Lin *et al.*, 2004 ▶; Shen *et al.*, 2004 ▶), where routine temperature measurements are often difficult. We will use the same principle to determine the energy scale *E*(ω) of a HRM. We assume that the sample temperature is accurately measured, *e.g.* to achieve a 0.1% error around room temperature then an accuracy of 0.3 K would be required. The mismatch of an assumed energy *E* relative to the true energy ω is quantified by a correction function ∊(ω), *i.e.* ∊(ω) = ω + ∊(ω). Also the elastic peak in the spectrum provides a true reference energy and therefore *E*(0) = 0 and ω(0) = 0. In practice, the corrections ∊ are expected to be small for a well aligned and controlled HRM. We can now rewrite the detailed-balance principle,
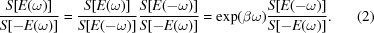
The ratio on the left is directly obtained from the measured data using the uncalibrated scale *E*(ω). With the relations *E*(−ω) = −*E*(ω) + ∊(ω) + ∊(−ω), ∊_+_(ω) = [∊(ω) + ∊(−ω)]/2 and ∊_−_(ω) = [∊(ω) − ∊(−ω)]/2, the right-hand side of (2) can be expressed as
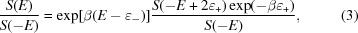
where the ω argument has now been omitted. If the energy correction function has only uneven terms, *i.e.* ∊_+_ = 0, the ratio on the right-hand side becomes 1, and we obtain an explicit expression for the mismatch. The lowest-order correction term is linear and therefore uneven. We will discuss this case in more detail.

In the linear-correction case the detailed-balance equation (3) simplifies to *S*(*E*) = *S*(−*E*) exp(ηβ*E*), where the energy correction is described by a constant scaling factor η. For measured data, the direct use of this expression is often not practical because each ratio can have substantial noise fluctuations. An improvement suggested earlier (Lin *et al.*, 2004 ▶; Shen *et al.*, 2004 ▶; Zhao *et al.*, 2004 ▶) is to solve the non-linear equation
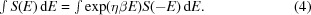
Also a standard optimization procedure such as
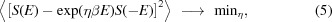
where 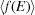 = 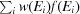 and *w*(*E*) is a weight function derived from the estimated statistical fluctuations of the measured data, provides good results for the value of η. The range for integration or summation would typically cover a region of the spectrum which provides the highest counting rates but excludes a small interval around the elastic scattering peak. Yet another method was suggested (Sturhahn & Jackson, 2007 ▶) to obtain an average temperature of the sample, or in our case an average scaling factor. It is convenient to define a thermal asymmetry function *A*(*E*) as follows, 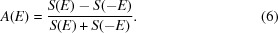
From the detailed-balance principle we expect the thermal asymmetry to be given by 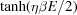, and the optimization procedure for η is formulated as
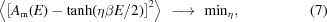
where *A*_m_(*E*) is calculated from the measured data. In case the energy calibration is already well established, the same minimization procedure can be used to determine the actual sample temperature 1/(*k*_B_ηβ), where 1/(*k*_B_β) is the initial guess. The methods represented by (4) and (7) have been implemented into the *PHOENIX* software package (Sturhahn, 2000 ▶) which is distributed under the *GNU* public license.

In practice, a quadratic correction of the energy scale may not be negligible, and we have to analyze (3) in more detail. The reasonable assumption of small energy corrections, *i.e.* 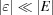 over the energy range to be calibrated, permits us to expand *S*(−*E* + 2∊_+_) in (3), and (6) then takes the form
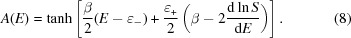
The appearance of the derivative of the measured spectrum is the direct result of a symmetric energy correction, and the corresponding features in the thermal asymmetry from measured data are distinct from the smooth 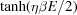 function. Now we introduce parameters ξ and υ *via* ∊_−_ = −ξ*E* and ∊_+_ = −ν*E* ^2^ and linearize (8) with respect to them,
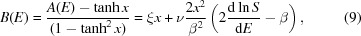
where *x* = β*E*/2. The optimization procedure for ξ and ν is formulated as
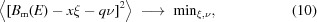
where *q* = 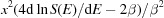. The optimal solutions of this linear least-square-root procedure are straightforward,
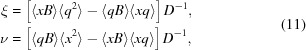
where *D* = 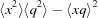.

## 3. Experiments and results

**Figure 1:**
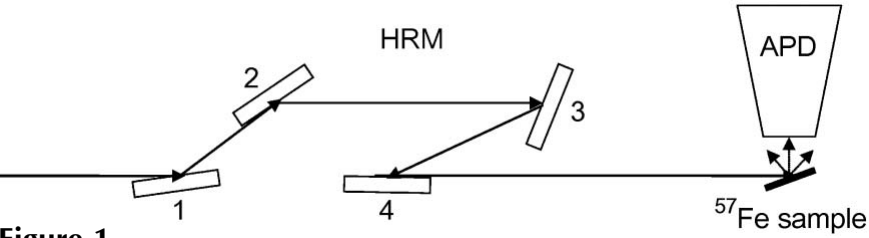
Experimental set-up for NRIXS measurement. HRM: high-resolution monochromator; APD: avalanche photodiode timing detector.

We performed a NRIXS measurement using the 14.412497 keV resonance of ^57^Fe to demonstrate our method. A schematic of the experimental set-up is shown in Fig. 1 ▶. The HRM produces 1.1 meV bandwidth X-rays with a spectral efficiency of 25%. It uses four flat asymmetrically cut silicon crystals with reflections (400), (400), (1064), (1064). Crystals 1 and 2 collimate the X-rays, and the relative angular position of crystals 2 and 3 defines the transmitted energy. The fourth crystal redirects the beam to the forward direction and restores size and divergence of the incident beam. The transmitted X-ray energy is changed by small rotations (typically less than 100 µrad) of the first pair of crystals (1 and 2) and the second pair of crystals (3 and 4). The temperatures of the individual crystals are monitored continuously with mK precision, and the angle positions are derived from the mechanical goniometer calibration. The transmitted X-ray energy relative to the nuclear resonance energy is then given by (Toellner *et al.*, 1997 ▶)
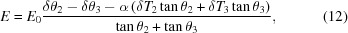
where *E*_0_ = 14.412497 keV is the nuclear transition energy of ^57^Fe, and α = 2.56 × 10^−6^ K^−1^ is the linear thermal expansion coefficient of silicon at room temperature. Bragg angles θ_*i*_ for the reflection of crystals *i* are calculated for energy *E*_0_ using the lattice constant of silicon at room temperature, 0.54310196 nm. The value of δθ_*i*_ describes the angular rotation of crystal *i* relative to its position at *E*_0_ which is identified by the presence of the elastic peak in the NRIXS spectrum, and δ*T*_*i*_ is the temperature change of crystal *i* with respect to its temperature when the HRM was tuned to the elastic peak and transmitted energy *E*_0_.

**Figure 2:**
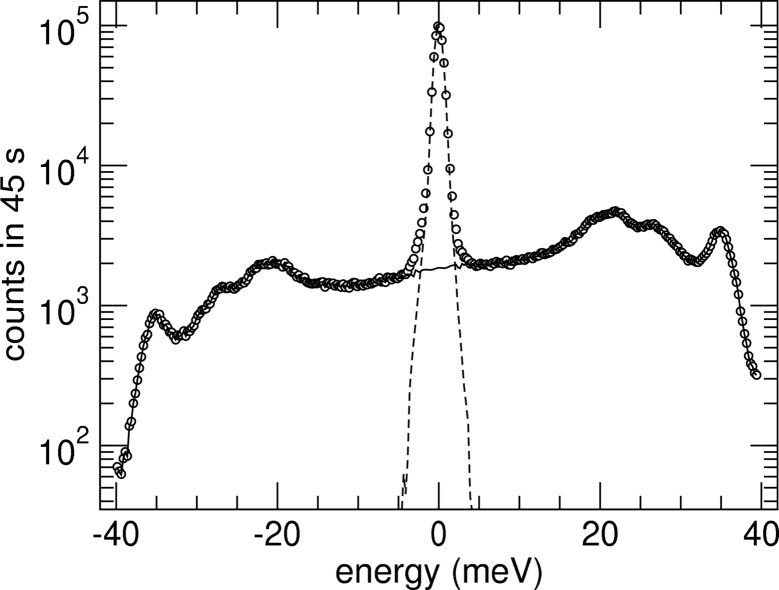
NRIXS spectrum of an iron foil under ambient conditions. Symbols shows the total counts of delayed X-ray photons *versus* the energy of the X-rays incident on the sample. Zero energy corresponds to the nuclear transition energy of 14.412497 keV. The dashed line shows the simultaneously measured resolution function of the monochromator. The solid line represents the inelastic part of the spectrum obtained by subtraction of the appropriately scaled resolution function from the data. The average background is 0.68 counts and significantly below the lowest signal.

The applications and technical aspects of NRIXS have been discussed in various publications (see, for example, Sturhahn & Jackson, 2007 ▶; Sturhahn, 2004 ▶; Röhlsberger, 2004 ▶; Chumakov & Sturhahn, 1999 ▶). We collected NRIXS data from a 50 µm-thick iron foil 95% enriched in the resonant ^57^Fe isotope at beamline 3-ID of the Advanced Photon Source. The ^57^Fe foil was chosen because it promises the highest signal of resonantly scattered X-rays. A calibrated thermometer measured the sample temperature which was stable at 298.0 (2) K. A total of 15 scans were collected. In each scan the HRM energy was tuned ±40 meV around the nuclear transition energy in 0.25 meV steps. After each step the nuclear resonant signal was collected for 3 s. For each scan the *E*_0_ positions were determined by the position of the elastic peak, and the energy scale was calculated according to (12). The nuclear resonant signals for the scans were then added and are shown in Fig. 2 ▶. The average detector noise which is energy independent was 0.015 counts s^−1^ and was quite small compared with the average signal of 44 counts s^−1^. The central peak at *E* = 0 is caused by elastic scattering from the sample, and its shape reflects the resolution function of the HRM. For positive energies the X-rays are too energetic to excite the nuclear resonance directly, and phonons must be created simultaneously. In the region of negative energies, the X-ray energy is too small, and phonons must be annihilated to produce resonant excitation. The presented calibration procedure is based on analysis of the asymmetry function (6), and therefore it is not necessary to measure the complete excitation spectrum. In general, parts of the spectrum with high intensity are preferable, but for a reliable determination of a quadratic energy correction the data should also include regions with well defined features. In the case of b.c.c.-Fe, ±40 meV was a suitable range.

**Figure 3:**
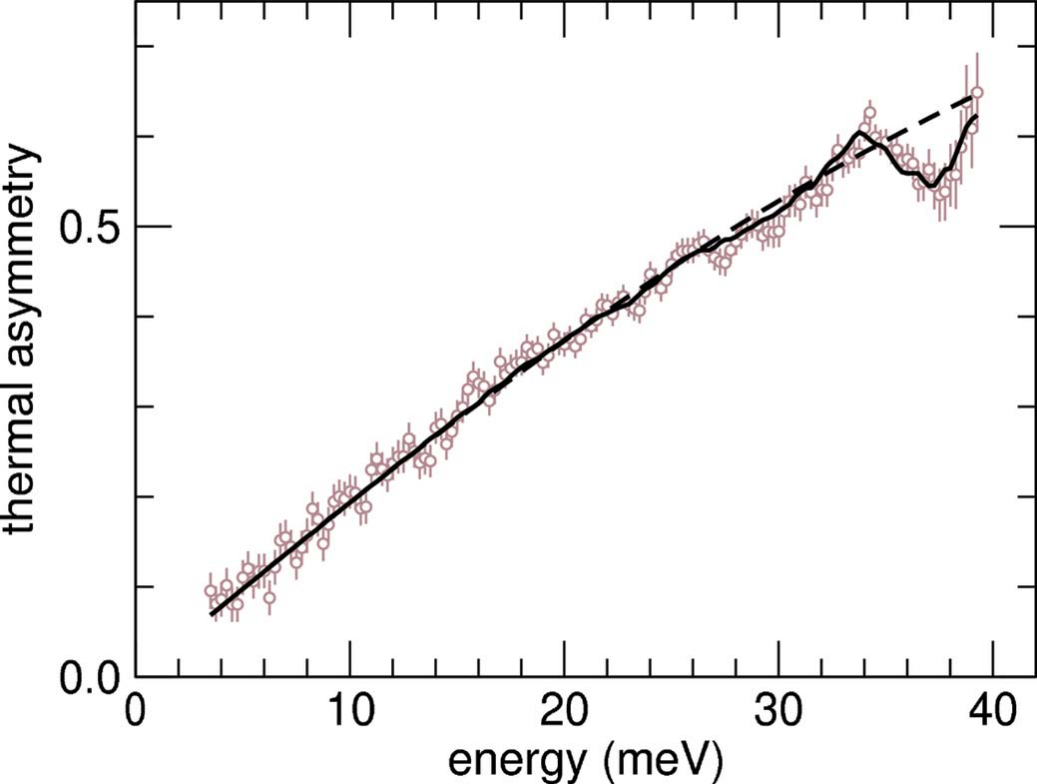
Thermal asymmetry function after (6) *versus* energy. The circles show values calculated directly from the measured data shown in Fig. 2 ▶, the solid line shows the result of a quadratic least-square procedure (10) with ξ = 0.0062 (34) and ν = 1.06 (9) × 10^−4^ meV^−1^, and the dashed line represents results of a linear least-square procedure (7) with η = 0.993 (5).

**Figure 4:**
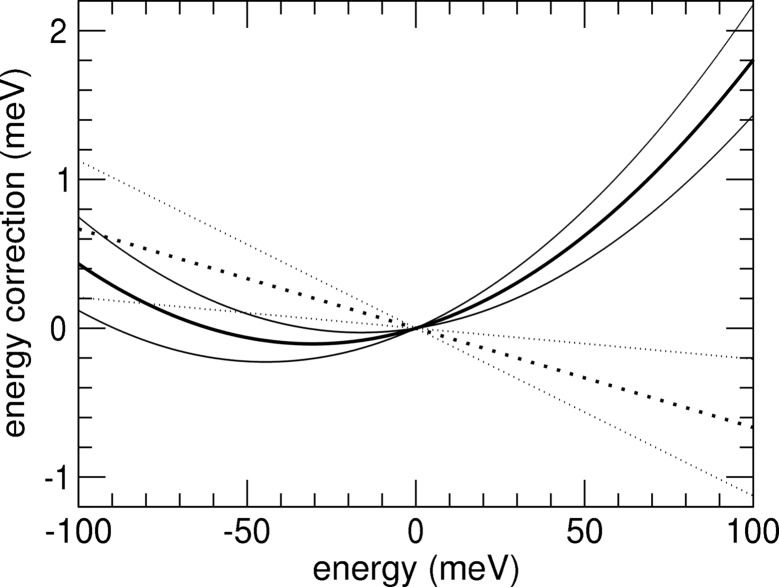
Energy correction and error margins *versus* energy. The solid and dotted lines represent results for quadratic and linear corrections, respectively.

After subtraction of the minor noise background from the NRIXS signal, the added data are simply proportional to the phonon excitation probability *S*(*E*)d*E* with the exception of the region dominated by the elastic peak where saturation effects become important (Sturhahn *et al.*, 1995 ▶). For the data in Fig. 2 ▶ the proportionality factor is 3.15 × 10^6^, and the uncertainty of the zero energy defined by the elastic peak is 0.003 meV. By using (7) in the energy interval between 5 meV and 40 meV, we obtain η = 0.993 (5) and therefore ω = 0.993 (5) *E* as the corrected energy scale. On the other hand, the addition of a quadratic term using (10) provides ξ = 0.0062 (34) and ν = 1.06 (9) × 10^−4^ meV^−1^ and therefore ω = 1.0062 (34) *E* + 0.000106 (9) *E* ^2^ meV^−1^. Fig. 3 ▶ displays results of both fit procedures. Clearly the region above ∼33 meV is much better reproduced using the quadratic correction, and we favor the latter over the scaling factor approach. The corrected energy scale and its error are then given by 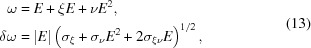
where the σ values are variances of the fit parameters. The energy correction ω − *E* and its error are shown in Fig. 4 ▶. The small energy corrections obtained indicate a very good control over the parameters entering (12). The error arises from counting statistics and puts the accuracy of this energy calibration at about 0.35% in the energy range of ±100 meV around the nuclear transition energy. The measured energy separation of two phonon peaks *E*_2_ − *E*_1_ experiences a correction of (*E*_2_ − *E*_1_)[ξ + ν(*E*_2_ + *E*_1_)] and an uncertainty of |*E*_2_ − *E*_1_|[σ_ξ_ + σ_ν_(*E*_1_ + *E*_2_)^2^ + 2σ_ξν_(*E*_1_ + *E*_2_)]^1/2^ where the variances are defined in (20). The relative error of the phonon peak separation is thus [σ_ξ_ + σ_ν_(*E*_1_ + *E*_2_)^2^ + 2σ_ξν_(*E*_1_ + *E*_2_)]^1/2^, and for close peaks with *E*_1_ ≃ *E*_2_ we obtain energy uncertainties of 2.5% and 4.8% for separated peaks around 50 meV and 100 meV, respectively.

## 4. Discussion

The formulations (7) and (10) show that the thermal asymmetry of the measured NRIXS spectrum plays a key role in the accuracy obtainable for the energy calibration. In the case of linear scaling, the variance of the fit parameter η can be estimated by
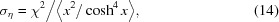
where χ^2^ is the normalized χ^2^ value of the least-square optimization, η is the optimum value for the calibration factor and *x* = β*E*/2. Temperature and statistical variations of measured data are the key factors that influence σ_η_. For a quantitative discussion of these effects, a few reasonable simplifications are made: the normalized χ^2^ value is set to 1; the optimum value for the calibration factor is set to 1; the weight function is estimated by the inverse variance of the asymmetry function (6) obtained with the measured signal
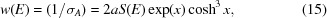
where *a* is the proportionality factor between the measured signal *I*(*E*) and the excitation probability density, *i.e.* *I*(*E*) = *a* *S*(*E*). The variance is then expressed as
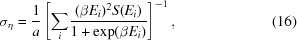
where the summation is over measured data points with positive energies *E*_*i*_. Clearly a smaller variance is obtained if the energy range of the measurement preferably covers regions with large values of *S*(*E*). Whereas the dependence of the variance on the proportionality factor is quite simple, the temperature behavior is more complex.

Our discussion of the temperature dependence of σ_η_ begins with the derivation of the asymptotic behavior. Very high (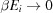) and very low (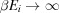) temperatures typically lead to large variances. In the first case we may approximate (16) by
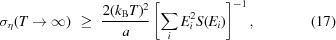
and we see that the error of η increases approximately linearly with temperature. This behavior is plausible because the contrast between Stokes and anti-Stokes side bands diminishes with increasing temperature. On the other hand, very low temperatures give the approximation
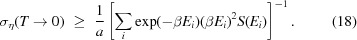
The exponential increase of the variance with inverse temperature is explained by the diminishing intensity of the anti-Stokes side bands with decreasing temperature. Both asymptotic values for the variance are unlimited, and thus there should be a minimum variance with temperature for a given material and data range.

**Figure 5:**
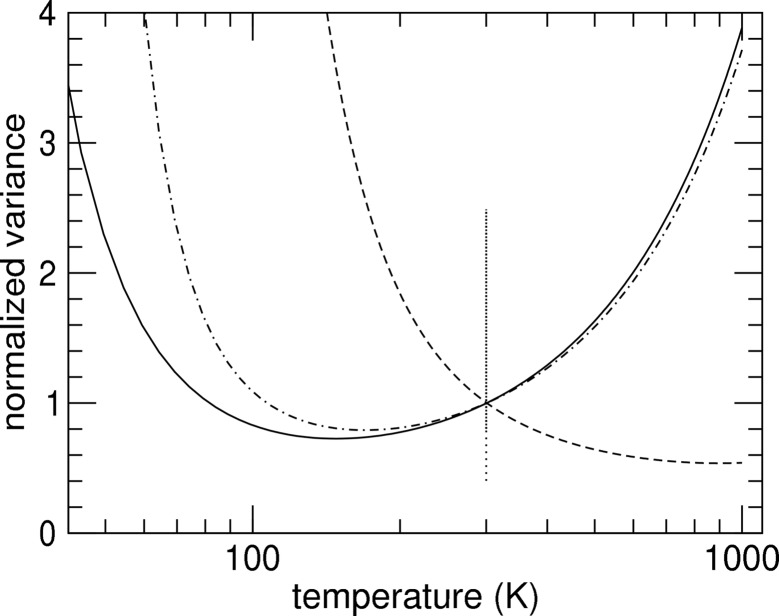
Normalized variances *versus* temperature. Solid and dashed lines show variances of linear and quadratic energy corrections, respectively. The dash-dotted line gives the variance of the energy correction at 50 meV. The dotted marker indicates the temperature in the NRIXS measurement. Variances are normalized to their values at 300 K.

The intrinsic temperature dependence of *S*(*E*) complicates a detailed study of (16). We calculated the phonon excitation probability from the phonon density-of-states of b.c.c.-Fe determined by NRIXS (Sturhahn, 2004 ▶) using the quasi-harmonic formalism described earlier (Sturhahn & Jackson, 2007 ▶; Chumakov & Sturhahn, 1999 ▶; Sturhahn & Kohn, 1999 ▶; Singwi & Sjölander, 1960 ▶),
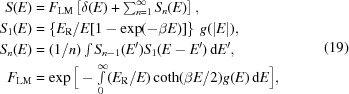
where *F*_LM_ is the Lamb–Mössbauer factor, *E*_R_ = 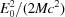 is the recoil energy (with nuclear transition energy *E*_0_, mass of the nuclear resonant isotope *M* and speed of light *c*), and *g*(*E*) is the partial phonon density-of-states normalized by 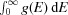 = 1. The value of 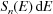 gives the probability for the simultaneous creation/annihilation of *n* phonons with a total energy between *E* and *E* + d*E*. In Fig. 5 ▶ we show calculated variances using (16) and (19) normalized to values at 300 K. The optimum temperature for a calibration procedure using linear scaling (7) is about 150 K for a b.c.c.-Fe sample. However, the improvements compared with room-temperature measurements are not substantial, and a low-temperature experiment would increase complexity.

In case a quadratic energy correction is needed, the variances of the fit parameters ξ and ν are estimated by 
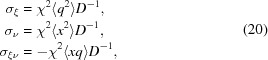
where σ_ξν_ expresses the correlation of the parameters ξ and ν, χ^2^ is the normalized χ^2^ value of the least-square optimization, and the abbreviations *x* = 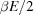, *q* = 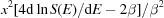 and *D* = 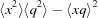 were used. For a quantitative discussion of the variances, we assume that the normalized χ^2^ value is 1, the values for ξ and ν are set to 0, and the weight function is estimated by the inverse variance of the function (7) obtained with the measured signal
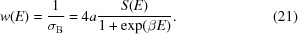
The variance of the linear correction parameter is then expressed as
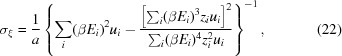
where 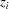 = 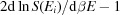, 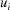 = 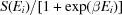, and the summation is over measured data points with positive energies 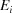. This variance is slightly larger than (16) owing to parameter correlation. The variance of the quadratic correction parameter is given by
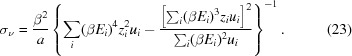
In contrast to σ_ξ_ the high-temperature limit for σ_ν_ is finite and given by
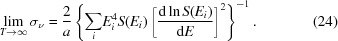
In fact, the determination of the quadratic correction of the energy scale does not rely solely on the detailed-balance principle but also on sharp features in the spectrum for which the derivative d*S*/d*E* becomes large. In our experiment this effect leads to a rather small relative error for ν of 8.5% whereas the relative error of ξ is a much higher 55%. In Fig. 5 ▶ we show calculated variances using (22), (23) and (19) normalized to values at 300 K. In this graph the values for σ_ξ_ and σ_η_ are indistinguishable and differ significantly from σ_ν_. Whereas the variance of the linear energy correction term assumes a minimum around 150 K, the quadratic energy correction becomes more accurate with increasing temperature. The variance of the energy correction (13) given by δ^2^ω/ω^2^ at 50 meV energy is also displayed in Fig. 5 ▶. The minimal uncertainty occurs around 170 K and is mostly determined by the variance of the linear correction term. In all simulations of this section and in the optimization procedures in the previous section, the lower limit of the summations is chosen to avoid spectral contributions from the tails of the elastic peak which was 3.5 meV for the presented data. The upper limit of about 40 meV for the summations is chosen to achieve best counting statistics but also to include the sharp drop-off near 35 meV. The scaling factor *a* is determined by the quality of the instrument, *i.e.* flux on the sample and efficiency of the time discrimination circuit, and the collection time.

**Figure 6:**
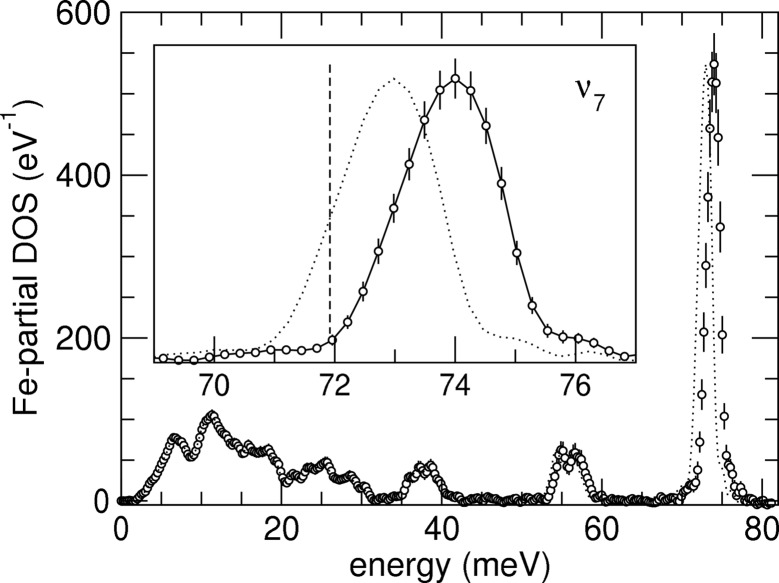
Fe-partial phonon density of states of K_2_MgFe(CN)_6_ at 30 K derived from NRIXS data. Symbols and dotted lines represent data with corrected and uncorrected energy scales, respectively. The inset shows part of the spectrum pertaining to the ν_7_ mode. The dashed marker indicates the line position from infrared spectroscopy (Nakagawa & Shimanouchi, 1962 ▶).

Fig. 6 ▶ shows the Fe-partial phonon density of states of K_2_MgFe(CN)_6_ at 30 K derived from NRIXS data. The octahedral metal cyanide complex K_2_MgFe(CN)_6_ has been intensively studied by infrared spectroscopy (Nakagawa & Shimanouchi, 1962 ▶), NRIXS (Chumakov *et al.*, 2003 ▶) and model calculations (Zakharieva-Pencheva & Dementiev, 1982 ▶), and the stable presence of high-energy excitation modes has often been used as energy scale calibrant in NRIXS experiments. In our data the ν_7_ mode, as assigned earlier (Nakagawa & Shimanouchi, 1962 ▶), is observed at 72.97 (6) meV. By applying energy scale correction and uncertainty (13), the ν_7_ mode shifts to 74.0 (3) meV or 596 (2) cm^−1^, which is slightly higher than the value of 72.5 meV or 585 cm^−1^ from infrared spectroscopy measurements at room temperature (Nakagawa & Shimanouchi, 1962 ▶). Also using the NRIXS technique but employing a different instrument, Chumakov *et al.* (2003 ▶) give a value of 74.3 meV for the ν_7_ mode at 30 K but do not provide an uncertainty. The agreement between these values is encouraging, but the deviation from the infrared value needs further explanation. Assuming that the K_2_MgFe(CN)_6_ samples used in the different experiments were of equal quality and specification, two considerations could reconcile the results: the effect of temperature on the ν_7_ mode and/or the effect of dispersion of the ν_7_ mode. The data shown in Fig. 6 ▶ give a width for the ν_7_ mode of 1.8 meV which is almost twice the energy resolution of the instrument. A plausible explanation of this broadening could be dispersion of the ν_7_ mode. Whereas infrared spectroscopy provides frequencies of vibrations very close to zero momentum transfer (the Γ point of the Brillouin zone), NRIXS spectra are integrated over momentum transfers and dispersion effects would potentially broaden and shift the ν_7_ peak.

We demonstrated the energy calibration using NRIXS spectra, but the same approach can be applied to non-resonant IXS. In that case, highest counting rates would most likely result from single crystals at suitably chosen orientation and appropriate momentum transfers. The option to use arbitrary momentum transfers would overcome some of the limitations intrinsic to energy calibration *via* Raman or infrared frequencies. However, for very large energy transfers, *e.g.* above 100 meV, counting rates decrease substantially and calibration by Raman or infrared spectra may remain the safest solution, particularly if the extrapolation of the energy correction (13) becomes uncertain.

## 5. Summary

Calibration of the energy scale is often a difficult procedure in high-resolution IXS measurements. In this paper we described a method that is based on the detailed-balance principle. We demonstrated an accuracy of 0.35% in an energy range of ±100 meV using NRIXS data. The data presented in Fig. 2 ▶ took only four hours to collect with minimal changes to the experimental set-up. The same procedure can be applied in momentum-resolved experiments and provides independent calibration in temperature scans of back-reflecting crystals in the instrument. The presented method is independent of sample properties, and the calibration procedure can be performed with samples selected for optimum scattering intensity and energy range. The decreasing intensity of phonon excitations with increasing energy transfer ultimately limits the applicability of the detailed-balance principle for accurate calibrations. In the example presented here, data were mostly limited to about the ±50 meV range and extrapolation to ±100 meV seems reasonable. However, the presence of a quadratic correction term may render extrapolation less reliable, and the availability of data at higher energies would be advantageous. Keeping these limitations in mind, we have presented a viable alternative to existing calibration methods for hard X-ray instruments in the ±100 meV range that is easy to implement in IXS experiments.
